# Bioremoval of tannins and heavy metals using immobilized tannase and biomass of *Aspergillus glaucus*

**DOI:** 10.1186/s12934-024-02477-z

**Published:** 2024-07-25

**Authors:** Moataza Mahmoud Saad, Abdelnaby Mahmoud Saad, Helmy Mohamed Hassan, Eman I. Ibrahim, Amany A. Hassabo, Basant A. Ali

**Affiliations:** Microbial Chemistry Department, National Research Centre (NRC), 33 EL-Bohouth St., Dokki 12622, Giza, Egypt

**Keywords:** Tannase, Gallic acid, *Aspergillus glaucus*, Immobilization, Heavy metals, Biosorption

## Abstract

**Background:**

The presence of inorganic pollutants and heavy metals in industrial effluents has become a serious threat and environmental issues. Fungi have a remarkable ability to exclude heavy metals from wastewater through biosorption in eco-friendly way. Tannase plays an important role in bioconversion of tannin, a major constituent of tannery effluent, to gallic acid which has great pharmaceutical applications. Therefore, the aim of the current study was to exploit the potential of tannase from *Aspergillus glaucus* and fungal biomass waste for the bioremediation of heavy metals and tannin.

**Results:**

Tannase from *A. glaucus* was partially purified 4.8-fold by ammonium sulfate precipitation (80%). The enzyme was optimally active at pH 5.0 and 40 °C and stable at this temperature for 1 h. Tannase showed high stability at different physiological conditions, displayed about 50% of its activity at 60 °C and pH range 5.0–6.0. Immobilization of tannase was carried out using methods such. as entrapment in Na-alginate and covalent binding to chitosan. The effects of Na-alginate concentrations on the beads formation and enzyme immobilization revealed that maximum immobilization efficiency (75%) was obtained with 3% Na-alginate. A potential reusability of the immobilized enzyme was showed through keeping 70% of its relative activity up to the fourth cycle. The best bioconversion efficiency of tannic acid to gallic acid by immobilized tannase was at 40 °C with tannic acid concentration up to 50 g/l. Moreover, bioremediation of heavy metal (Cr^3+^, Pb^2+^, Cu^2+^, Fe^3+^, and Mn^2+^) from aqueous solution using *A. glaucus* biomass waste was achieved with uptake percentage of (37.20, 60.30, 55.27, 79.03 and 21.13 respectively). The biomass was successfully used repeatedly for removing Cr^3+^ after using desorbing agent (0.1 N HCl) for three cycles.

**Conclusion:**

These results shed the light on the potential use of tannase from locally isolated *A. glaucus* in the bioremediation of industrial tanneries contained heavy metals and tannin.

## Introduction

Tannery effluents having a high content of tannins, mainly polyphenols, represent a serious problem in pollution. Such pollutants can be enzymatically degraded and removed by tannases through hydrolysis of ester bonds of these polyphenols avoiding their polymerization [1, 2]. Beside effluents, tannins are also present in a variety of sources including tea, coffee, spices, as well as in various fruits and vegetables such as apricots, pomegranates, grapes, dates, eggplants, and strawberries, and in nuts like peanuts [3].

Tannin acyl hydrolase (TAH, E.C.3.1.1.20) is an enzyme that involved degrading pathway of tannin and thus has a huge industrial importance. TAH, commonly called tannase, catalyzes the hydrolysis of the central and terminal ester bonds between two aromatic rings of digallate in hydrolyzable tannins, gallic acid-based esters and other tannins, releasing gallic acid and glucose as the end products [4–7]. Fungi are a major source of valuable hydrolytic enzymes as well as economical byproducts which are needed for different modern industrial and medical applications [6, 8]. Different fungi have been reported as tannase producers such as *Aspergillus* species [9, 10] and *Penicillium* species [11].

Application of tannases reached to the food, beverages, chemical and pharmaceutical industries [12]. For instance, tannase is used in elaboration of tea powder and synthesis of gallic acid which is used in the industry of the antibiotic trimethoprim [8, 13–15]. Similarly, tannases have a wide applicability in laundry, detergent and leather industries as homogenizing agent for preparation of high-grade leather tannins [16–18]. However, the application of enzymes in industry remains limited due to the high cost of the bioprocess as well as the enzyme reusability versus stability.

The tannery industry is classified as one of the most polluting industries, generating large amounts of environmentally harmful liquids through all stages of the industrial process [19]. The presence of inorganic pollutants and heavy metals in industrial effluents particularly from electroplating, paint and tanning industries is become major environmental issues. Tannery effluents are highly complex, dark brown in color due to the presence of tanneries and have high organic content that vary according to the chemicals used. Tanneries release a large amounts of waste water containing chromium, ammonium, sulphate, dyes, and tannins. Chromium salts used in tanning process generates trivalent chromium which is toxic and carcinogenic due to its high solubility in water, permeability through biological membrane interaction with proteins and nucleic acids [20, 21]. Tannins toxicity is typically correlated with ingestion rates and absorbed tannins have the potential to cause liver and kidney necrosis. The hydrolysis of tannins by tannase is a cheap treatment to reduce the degree of toxicity of the effluents from the tannery. Similarly, industrial effluents from agrochemical industries have high levels toxic, carcinogenic and reactive pollutants such as heavy metals [22]. The discharge of industrial wastewater into water bodies will expose the environment and living entities such as plants, animals and humans to the harmful heavy metals [23]. Therefore, study of the removal of such polluting heavy metals in an environment-friendly way is urgently needed. Bio-adsorption approaches of heavy metals to the surface of the fungal biomass as biosorbents are effective environmentally friendly technology [24]. Biosorption has many advantages, such as using cheap biomass (live and dead bacteria or fungi) for removal of heavy metals and low cost treatment of large volume of effluents [25]. These heterotrophic microorganisms have the ability to form mycelial network and exploit the pollutant and use it as nutrients, which make the potential in bioremediation. Distinction of the fungal biomass as low-cost adsorbent is due to their large surface area and effective surface interaction [26]. Particularly, fungi can eliminate heavy metals by different means such as ion exchange or metal ion adsorption by the functional groups on the cell wall of the fungus. Mechanism of heavy metal biosorption starts by passive binding of fungal biomass to the metal ions and involves single or combination of different processes such as ion exchange, coordination, chelation, adsorption, complexation and electrostatic interaction [27–29]. Many of the fermentation processes end up by huge mass of fungal waste which in turn can be used as bio-adsorbent for the heavy metals [30, 31]. Fungi can also be easily grown in large quantities using simple fermentation methods and low-cost growth media. Therefore, the economical removal of metal ions from industrial effluents may be achieved by fungal biomass. Aftab et al. (2014) reported by *Aspergillus caespitosus* immobilized calcium alginate beads effectively removed lead (93% removal efficiency) from real wastewater from the paint industry [32]. Chaudhary et al., (2019) studied the effectiveness of *Aspergillus fumigates* in the bioremediation of tannin and chromium in tannery wastewater. They reported that *A. fumigates* had a maximum Cr removal efficiency of 65.1% [20].

Immobilization of enzymes via immobilization techniques such as adsorption, entrapment and covalent binding is the main strategy to improve enzyme functional properties such as activity and enzyme shelf life [33–35]. Currently, enzyme immobilization is preferred than using free enzymes due to long enzyme shelf life, stability under extreme conditions and multiple reuse for long periods. There are different available organic and inorganic carriers used for enzyme immobilization to ensure the highest retention of enzyme activity and stability, such as polymers, biopolymers. Alginate is a polysaccharide consists of glucuronic acid and mannuronic acid moieties, which has been successfully used for encapsulation of many biological molecules due to its biocompatibility and processivity [36]. Chitosan is a biodegradable and non-toxic natural polysaccharide which has antimicrobial potential [37]. Moreover, chitosan is considered as a suitable support for preparation of immobilized enzyme resistant to chemical degradation or metal ions disturbance.

The aim of this study is the potential use of tannase from a locally isolated fungus *A. glaucus* in bioremediation of tannery effluents containing tannins and/or heavy metals. Optimal physicochemical parameters affecting the activity of the partially purified tannase were investigated. Moreover, enzyme reusability in bioconversion of tannins to gallic acid as well as usability of *A. glaucus* biomass waste in removal of heavy metals in tannery effluents were addressed as value-added bioprocess.

## Methods

### Chemicals

Gallic acid methyl ester (methyl gallate) was purchased from Tokyo Chemical Industry TCI - Co., Gallic acid was obtained from oxford laboratory, tannic acid was purchased from AVI-CHEM., India, and Rhodanine was purchased from Sigma Chemical Co., U.S.A. All other chemicals used were of the highest analytical grade.

### Microorganism and culture conditions

**Fig. 1:**
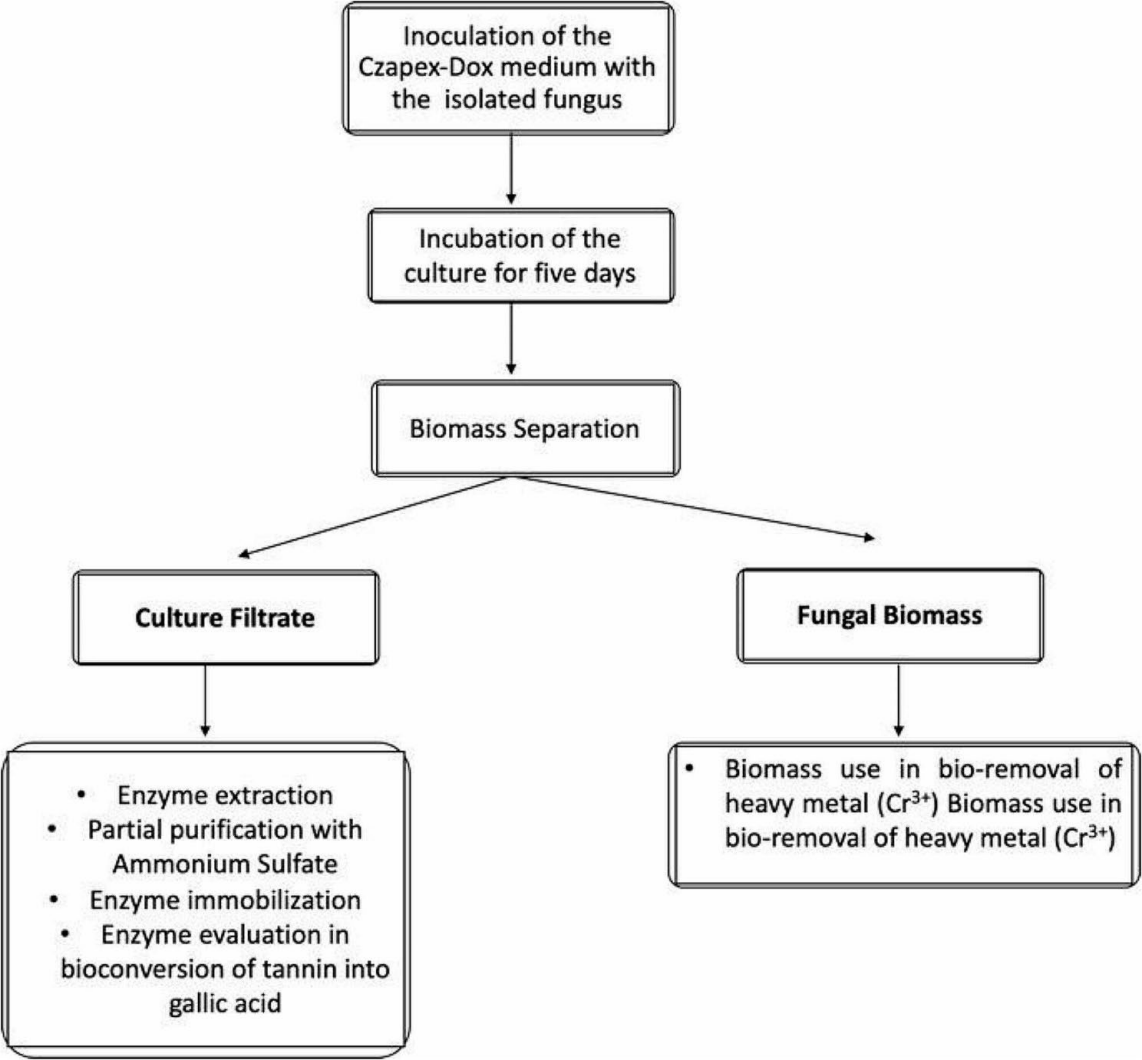
Workflow of tannase enzyme production and biomass production from *A. glaucus*

Tannase producing fungus used in this study was previously isolated from residual tea and identified as *A. glaucus* (GenBank accession number: MH205732) [38]. Figure 1 represents the work flow of tannase production, biomass preparation from *A. glaucus* and their evaluation in tannin bioconversion and heavy metal removal. For inoculum preparation; 1 ml of spore suspension of *A. glaucus* was inoculated in 250 ml Erlenmeyer flasks containing 25 ml of Czapek-Dox medium and incubated at 30 °C. *A. glaucus* was grown in previously optimized Czapex-Dox medium containing (g/l); NaNO_3_, 2; KCl, 0.5; MgSO_4_.7H_2_O, 0.5; KH_2_PO_4_, 1.0; FeSO_4_.7H_2_O, 0.01 and tannic acid, 10, adjusted to pH 5.5, with the moisture content of 75%. Spore suspension (1 × 10^6^ spores/ml) were used to inoculate the production medium and then incubated at 28 °C for five days.

### Enzyme extraction

Crude extract was extracted from the SSF-broth to obtain the extracellular tannase by adding 50 ml of citrate buffer (0.05 M, pH 5.0) to the SSF-broth. The flask was then maintained on rotary shaker at 180 rpm and 25 °C for 1 h. The culture mixture was then squeezed using a medical gauze followed by centrifugation at 11,200 rcf, 4 °C for 15 min to separate the crude extract. The clear part was used for tannase assay [39, 40].

### Tannase assay

Briefly, the reaction mixture consisted of 50 µl of diluted enzyme in 100 mM sodium-phosphate buffer pH 5.0 and 50 µl of 12.5 mM methyl gallate. The reaction mixture was incubated at 30 °C and at 600 rpm for 10 min. To stop the enzymatic reaction, 60 µl of 0.667% (w/v) methanolic rhodanine solution was added to the reaction mixture and left at 25 °C for 5 min. Then, 40 µl of 500 mM KOH was added to the reaction mixture and left for 5 min. Finally, 800 µl of distilled water was added and the mixture was incubated at 25 °C for 10 min before absorbance measurement at 520 nm. One unit of tannase activity is defined as the amount of the enzyme releasing one µmole of gallic acid per minute under assay conditions.

### Estimation of protein content

The protein content of the samples was determined according to the method of Bradford [41] using bovine serum albumin as a standard.

### Partial purification of tannase from *A. glaucus*

#### Ammonium sulfate precipitation

Ammonium sulfate was added to the crude enzyme to reach a saturation of 80% with constant stirring at 4 °C. Subsequently, the precipitate was then collected by centrifugation at 11,200 rcf at 4 °C for 15 min. The protein was resuspended in minimal amount of citrate buffer (0.1 M, pH 5) and dialyzed against the same buffer for 24 h at 4 °C. The enzyme activity and protein content of the fractions were measured. This precipitation process was continued up to 100% ammonium sulfate saturation.

### Biochemical characterization of the tannase from *A. glaucus*

#### Effect of pH on enzyme activity and stability

To determine the optimum pH for enzyme activity, the reaction mixture was carried out within the pH range of 3.0 to 8.0 using sodium citrate buffer (0.1 M, pH 3–5) and phosphate buffer (0.1 M, pH 6–8). For pH stability studies, enzyme preparations were incubated at various pH values [3–8] for 1 h before measuring the residual enzyme activity under standard assay conditions.

#### Optimum temperature and thermal stability

The optimal temperature for tannase activity was determined by carrying out the enzyme reaction at different temperatures from 30 °C to 70 °C under optimal assay conditions. The thermal stability of tannase was studied by measuring the residual enzyme activity after incubating the enzyme at the same temperatures (30–70 °C) for 1 h.

#### Effect of metal ions on enzyme activity

To determine the effect of metal ions on the tannase activity, the enzyme solutions were incubated in the assay buffer containing 10 mM of metal salts (CaCl_2_, CuSO_4_, FeCl_2_, MgCl_2_, ZnCl_2_, CoCl_2_ and CrCl_3_, EDTA and β-mercaptoethanol) for 1 h at room temperature. Then the residual enzyme activity was measured under the standard assay conditions.

#### Immobilization of *A. glaucus* tannase

Immobilization of *A. glaucus* tannase by the entrapment method using sodium alginate was addressed [42]. Briefly, appropriate volume of partially purified enzyme was mixed with the same volume of sodium alginate solution at different concentrations (3–6%). The obtained mixture was extruded dropwise into 0.2 M CaCl_2_ solution with shaking. Subsequently, the formed beads were allowed to harden for 1 h, after which they were washed with 50 mM sterile citrate buffer, pH 5.0 and preserved in CaCl_2_ solution at 4 °C until further use.

Additionally, the partially purified tannase was covalently immobilized on chitosan, which was activated by 2.5% (v/v) glutaraldehyde [43]. Briefly, one gram of chitosan was dissolved in 100 ml of HCl containing glutaraldehyde at 30 °C for 2 h. Precipitation of the solubilized chitosan was achieved using 1 M NaOH. The precipitate was obtained by filtration and washed twice with distilled water to remove excess glutaraldehyde. Immobilization of tannase was performed by adding one ml of an enzyme solution in citrate buffer (50 mM, pH 5.0) to 5.0 mg of chitosan. The mixture was slowly stirred for 1 h at 30 °C. After that, the solids were separated, washed with the same buffer and tested for enzymatic activity. The immobilization efficiency was expressed by the ratio of specific activity of immobilized enzyme to the free one.

$$\eqalign{& Immobilization \,efficiency \left( \% \right){\rm{ = }} \cr & \left[ {{\matrix{{\rm{Specific}}\>{\rm{activity}}\>{\rm{of}} \hfill \cr {\rm{the}}\>{\rm{immobilized}}\>{\rm{enzyme}} \hfill \cr} \over \matrix{{\rm{Specific}}\>{\rm{activity}}\>{\rm{of}} \hfill \cr {\rm{the}}\>{\rm{free}}\>{\rm{enzyme}} \hfill \cr \>({\rm{non}} - {\rm{immobilized}}\>{\rm{enzyme}}) \hfill \cr} }} \right] \times 100 \cr}$$

, where the specific activity (U/mg) of the free enzyme = 5.55.

### Scanning microscopy (SEM) analysis of the immobilized beads

The surface of the sodium alginate beads modified by immobilization of *A. glaucus* tannase was visualized using scanning microscopy technique (SEM) at 20 kV accelerating voltage. Na alginate beads was used as control. All samples were coated with gold (Sigma-Aldrich, USA) and visualized using QUANTA FEG250 (Eindhoven, The Netherlands). The magnification X500 and X2000 were used to examine the surface of the immobilized beads compared to the control. Separate areas were investigated in order to check the possible changes in the immobilized sample versus the control.

### FT-IR analysis of the immobilized beads

The FT-IR analysis was performed using Vertex 70 spectroscope (Bruker, MA, USA) equipped with ATR-diamond crystal within range of 4000 –370 cm with 1 cm resolution. For samples preparation, all samples were dried in exicator for 12 h to remove the excess water. OPUS 7.0 program (bruker, MA, USA) was used to analyze the spectra.

### Bioconversion of tannins by tannase

Tannase from *A. glaucus* was incubated in the different tannic acid solutions with different concentrations (50 g/l, 100 g/l and 200 g/l), pH 5.0 at 28 °C under shaken conditions. Gallic acid and residual tannic acid were estimated in all samples [44]. Spectrophotometric estimation of gallic acid was carried out by the method described by Sharma et al. (2000) [45]. A 300 µl of methanolic rhodanine (0.667% in methanol) was added to the standard gallic acid or suitably diluted sample followed by addition of 200 µl of 0.5 M potassium hydroxide. After incubation at 300 °C for 5 min., 4 ml distilled water was added. The absorbance was read at 520 nm after 10 min. The total tannin content of each sample was determined according to the method described by Abd-Elmotey et al. [46]. Briefly, the assay involved mixing 0.5 ml of the sample, diluted tenfold, with 2.5 ml of Folin-Ciocalteu’s phenol reagent, also diluted tenfold. After a 5-minute incubation, 2 ml of 20% (w/v) Na2CO3 was added, and the mixture was incubated for an additional hour at room temperature. Absorbance was measured at 760 nm. Percentage of tannin degradation was calculated according to the following formula:

$$\eqalign{& Percentage \,of \,tannin \,degradation{\rm{ }}\left( \% \right){\rm{ }} = \cr & \left[ {{{{\rm{Tannin}}\>{\rm{before}}\>{\rm{degradation}} - \>{\rm{Tannin}}\>{\rm{after}}\>{\rm{degradation}}} \over {{\rm{Tannin}}\>{\rm{before}}\>{\rm{degradation}}}}} \right] \times \>100 \cr}$$

### Evaluation of *A. glaucus* biomass waste in bioremediation of some heavy metal ions from aqueous solution

#### Heavy metal ions uptake by *A. glaucus* biomass

All bio-removal experiments were performed as batch experiments in which five grams of live fungal biomass (as a bio-removal agent) was added into 250 ml Erlenmeyer flasks individually containing 50 ml of metal ion solution (Cr^3+^, Pb^2+^, Cu^2+^, Fe^3+^ and Mn^2+^) and incubated in a shaking incubator at 150 rpm for 10 min. at 30 °C [47]. Similarly, 50 ml of metal(s) solution was incubated without biomass as negative control. Stock metal solutions were prepared as 1000 ppm using chloride salts; CrCl_3_, PbCl_2_, CuCl_2_, FeCl_3_ and MnCl_2_ (Merck (NJ, USA) or VWR chemicals BDH (PA, USA)). The biomass was harvested by filtration on filter paper (Whatman No. 1). The residual metal concentration in filtrate was analyzed for the determination of residual metal(s) by Flame Atomic Absorption Spectrophotometer (Agilent Technologies 200 series AA). For sample digestion, 10 ml of HNO3 (1:1) was mixed with the sample. The slurry was then heated at 95 °C and refluxed for 10 min. Sorption of metal ions by the biomass (metal ion uptake %) was calculated using the following expression;

$$\:Unsorbed\:metal\:\left(residual\:metal\:\%\right)=\left[\raisebox{1ex}{$\left(\text{A}\times\:100\right)$}\!\left/\:\!\raisebox{-1ex}{$\text{B}$}\right.\right]$$

$$\:Sorbed\:metal\:\%=\left[\raisebox{1ex}{$\left(100-A\right)\times\:\:100$}\!\left/\:\!\raisebox{-1ex}{$\text{B}$}\right.\right]$$

A = Metal ion concentration in supernatant.

B = Initial metal ion concentration (control).

### Effect of live and dead fungal biomass on Cr^3+^ uptake

Fungal biomass, including both live and dead forms, was assessed for its Cr3 + uptake capacity. Accordingly, five grams of wet fungal biomass, equivalent to 0.44 g dry weight, were subjected to pre-treatment before being incubated with a Cr3 + solution (concentration of 30 ppm at pH 5) under two separate conditions. The first treatment was boiling of fungal biomass in distilled water for 10 min the second one was soaking of fungal biomass in 5% KOH solution for 10 min. The fungal biomass was then separated by filtration, washed with 1 N HCl and thoroughly washed several times with distilled water until neutralization [47]. The living fungal biomass (untreated) was served as control.

### Cr^3+^ desorption and reloading of fungal biomass

Sorption of Cr^3+^ was carried out as described previously. After initial Cr^3+^ uptake, the fungal biomass was separated by filtration and repeatedly washed with distilled water. The metal ions content in the supernatant was determined. Sorbed metal ion by the biomass was taken as 100% for the subsequent uptake cycle. The biomass obtained from the initial biosorption (described above) was suspended in 50 ml of the desorbing agent (0.1 N HCl) for 1 h. The biomass was separated by filtration, washed and re-suspended in Cr^3+^ solution for 1 h. Similarly, 50 ml of the desorbing solution without the fungal biomass was considered as a negative control. By the end of incubation period, the biomass was separated and Cr^3+^ content in the clear filtrate was determined. The percentage of elution efficiency of the desorbing agent was calculated as follows:

$$\eqalign{& Elution \,efficiency{\rm{ }}\% {\rm{ }} = \cr & \left[ {{{{\rm{amount}}\>{\rm{of}}\>{\rm{desorbed}}\>{\rm{metal}}\>{\rm{ion}}} \over {{\rm{amount}}\>{\rm{of}}\>{\rm{biomass}}\>{\rm{used}}\>{\rm{for}}\>{\rm{metal}}\>{\rm{ion}}\>{\rm{sorption}}}}} \right] \times \>10 \cr}$$

### Statistical analysis

All experiments were performed in triplicates and results were presented as mean values ± standard error (SE).

## Results

### Partial purification of tannase from *A. glaucus*

Tannase from *A. glaucus* was partially purified 4.83-fold with a yield of 66.67% by fractional precipitation by ammonium sulfate (80%). The specific activity of the enzyme showed substantial increase from 4.09 to 19.76 U/mg protein.

### Biochemical characterization of tannase

#### Effect of pH on the activity and stability of tannase

**Fig. 2:**
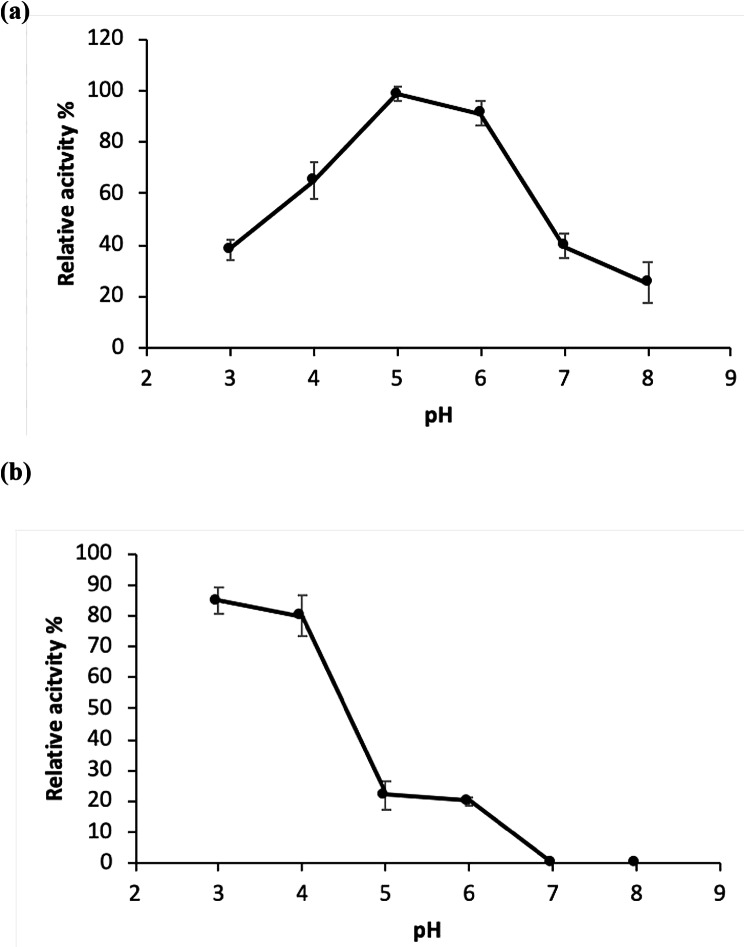
Effect of pH on the enzyme activity (**a**) and stability (**b**) of the tannase

The optimal pH for tannase activity was determined by measuring the enzyme activity at various pH values. As shown in Fig. (2a), the enzyme was most active at the pH range 4.0–6.0 with an optimal activity at pH 5.0. On the other hand, a sharp decrease in enzyme activity was observed above pH 6.0 indicating the acidic nature of the enzyme. Concerning pH stability of tannase, results showed that the enzyme was completely stable at pH range from 5.0 to 6.0 since no loss of enzyme activity was observed after 12 h of incubation (Fig. 2b). On the other hand, the enzyme was more stable at low pH values than at high pH values retaining about 38% of the maximum activity at pH 3.0, while the enzyme completely lost its activity at pH 8.0.

### Effect of temperature on the activity and stability of tannase

**Fig. 3:**
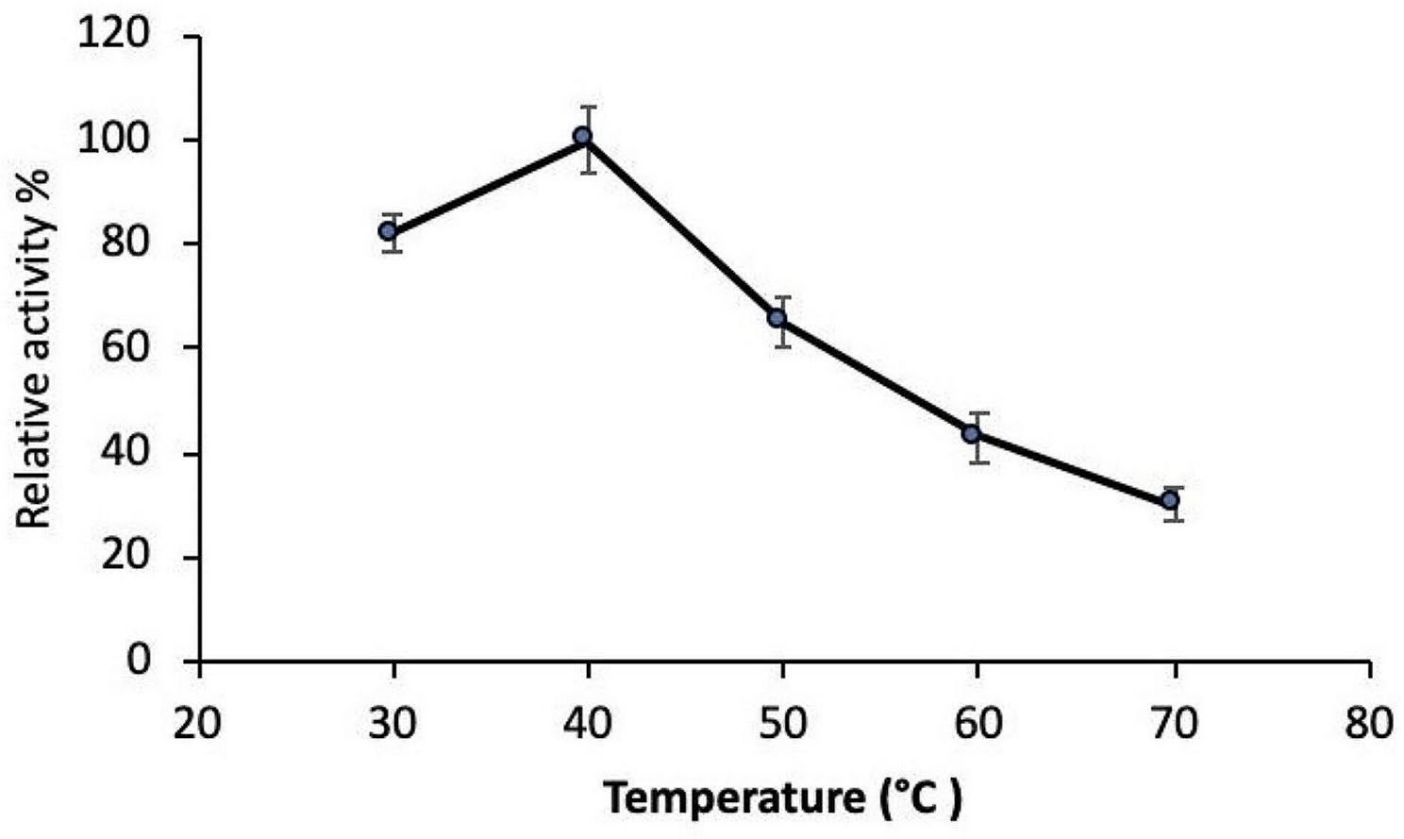
The effect of temperature on the tannase activity

**Fig. 4:**
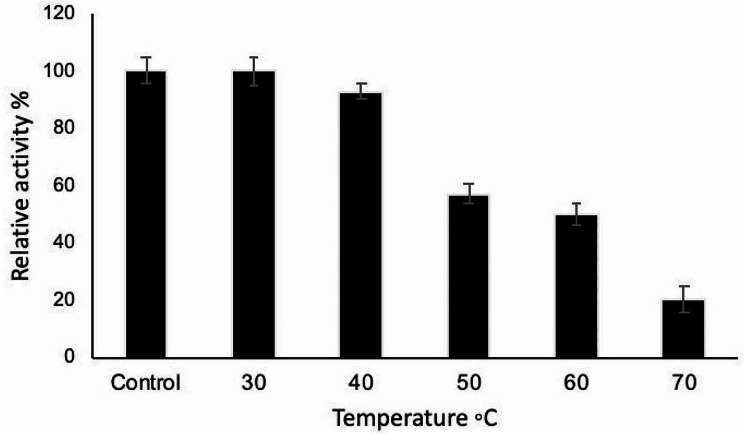
Thermal stability of tannase enzyme

Studies on the effect of temperature on the activity and stability of tannase revealed that the highest activity was recorded at the temperature range 30 –40 °C (Fig. 3). In addition, the thermal stability of tannase indicated that the enzyme was completely stable up to 40 °C, since no loss in catalytic activity occurred after 1 h of incubation (Fig. 4). Moreover, the enzyme still retained about 50% of its original activity after incubation at 60 °C for 1 h.

### Effect of metal ions and other chemicals on tannase activity

A study on the effect of different metal ions on tannase activity indicated that the enzyme was active in the presence of all metal ions tested except Fe^2+^, Cu^2+^ and EDTA (Table 1). On the other hand, β-mercaptoethanol decreased the enzyme activity the most due to disruption of the formation of disulfide bonds.

**Table 1:** Effect of metal ions on tannase activity

Metal ions & chemicals	Relative activity%
Control	100 ± 0.90
CrCl_3_	90.21 ± 1.06
MgCl_2_	83.08 ± 0.44
CaCl_2_	92.30 ± 0.61
CoCl_2_	70.77 ± 0.58
FeCl_2_	49.23 ± 0.43
CuSO_4_	64.62 ± 0.55
ZnCl_2_	80 ± 0.44
EDTA	56.92 ± 0.41
β-mercaptoethanol	46.15 ± 0.20

### Immobilization of *A. glaucus* tannase

The partially purified tannase was immobilized by two different methods, covalent binding using chitosan and Na-alginate entrapment method. Results showed that, the enzyme immobilized on Na-alginate exhibited higher specific activity in comparison with the enzyme immobilized on chitosan. Whereas the immobilization efficiencies of *A. glaucus* tannase immobilized on Na-alginate and chitosan were found to be 75% and 57% respectively (Table 2). In addition, Entrapment of tannase in Na-alginate resulted in 3–4 mm compact beads. It was observed that, the concentration of Na-alginate in the initial solution affect the bead properties. The beads prepared with 3% Na-alginate were fragile and loosely formed while those prepared with 4, 5 and 6% Na-alginate were non-fragile and compact beads. In addition, the enzyme beads prepared with 3% and 4% Na-alginate concentration showed higher activity (Sp. activity 4.16 and 3.84 U/mg, respectively) than enzyme beads of 5% and 6% Na-alginate (Sp. activity 1.28 and 0.32 U/mg, respectively). While a higher polymer concentration in beads can enhance stability, it may also restrict enzyme diffusion, leading to a reduced reaction rate. The better immobilization system was chosen according to the better enzyme stability, reusability and rate of catalysis. Therefore, 3% Na-alginate was finalized as the best immobilization efficiency comparing to other matrices.

**Table 2:** Immobilization of *A. glaucus* tannase

Immobilization	Activity of immobilized enzyme (U/ml)	Specific activity (U/mg)	Immobilization efficiency (%)
Entrapment Na alginate (3%)	39.12 ± 1.95	4.16 ± 0.32	75 ± 1.29
Entrapment Na alginate (4%)	36.88 ± 1.94	3.84 ± 0.48	69 ± 1.35
Entrapment Na alginate (5%)	18.96 ± 1.88	1.28 ± 0.14	23 ± 0.61
Entrapment Na alginate (6%)	10.97 ± 0.62	0.32 ± 0.05	5.70 ± 0.43
Covalent binding Chitosan	22.40 ± 0.93	3.2 ± 0.26	57 ± 2.61

### Examination of the surface of na alginate beads carrying tannase using the SEM Microscopy

The study of the surface of different materials used for the immobilization of enzymes requires modern tools such as scanned electron microscope (SEM).

**Fig. 5:**
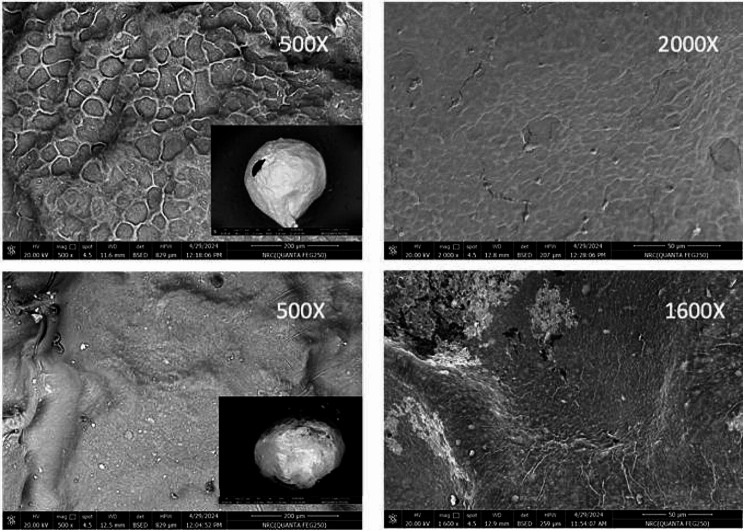
SEM micrographs of the surface of 3% Na alginate beads (control) **(Upper)**, SEM micrographs of the surface of 3% Na alginate beads containing tannase enzyme isolated from *A. glaucus*** (Lower)**

The microscopic analysis of the surface of Na alginate beads (3%) showed morphological changes among different analyzed samples (Fig. 5). Na alginate beads was used as control (Fig. 5a) while the sample of the Na alginate carrying the *A. glaucus* tannase (Fig. 5b). Interestingly, the SEM micrographs revealed that the immobilization of tannase into the composition of the Na alginate beads resulted in smoothing of the biochemical matrix of the beads compared to the control. This observation suggests an enhancement of the mechanical properties of the immobilized sample.

### Examination of the surface of na alginate beads carrying tannase using FT-IR

**Fig. 6:**
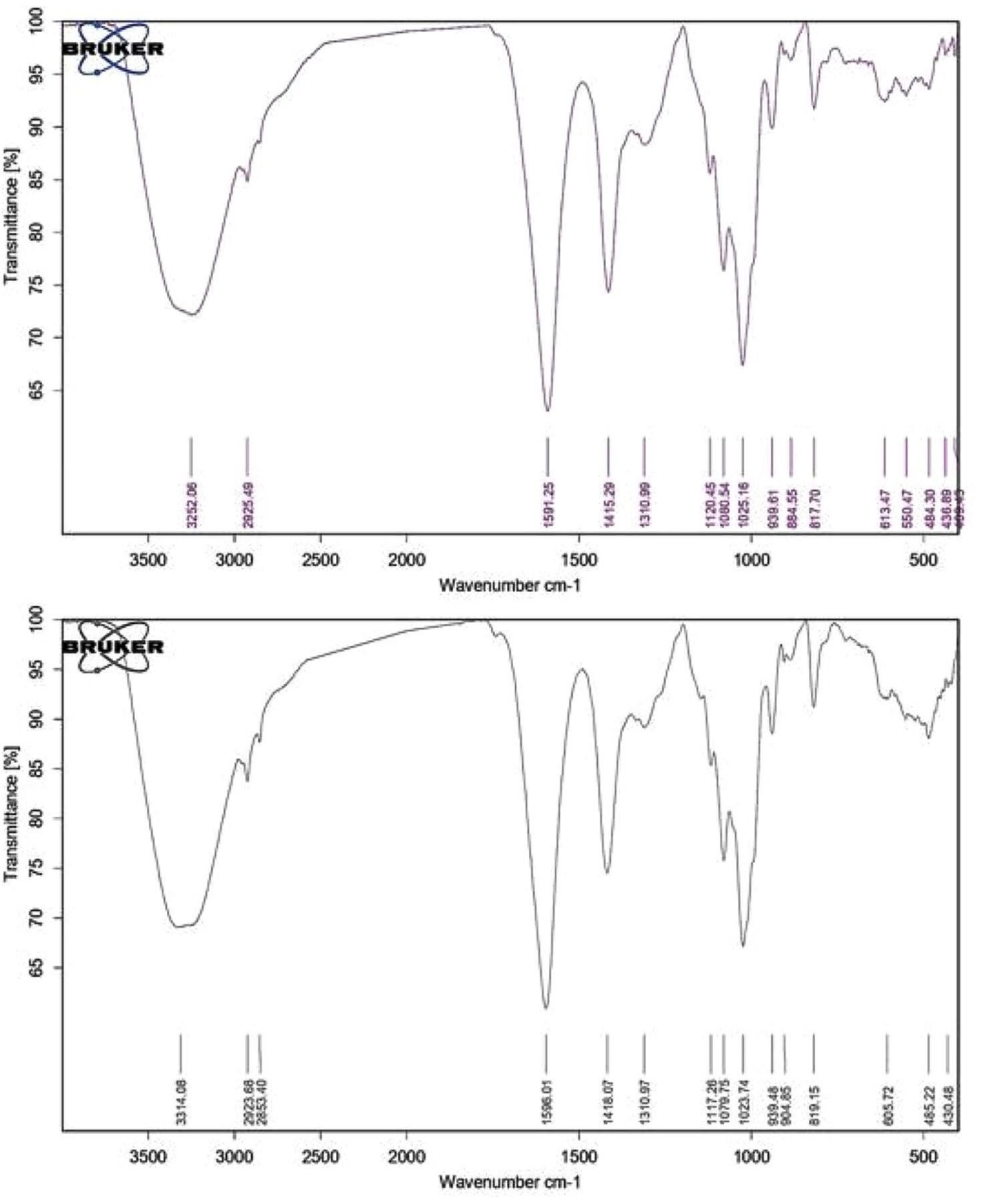
FT-IR spectra of 3% Na alginate beads (control) **(Upper)**, FT-IR spectra of 3% Na alginate beads containing tannase enzyme isolated from *A. glaucus*** (Lower)**

Infrared Spectroscopy was performed for both free Na alginate beads and beads containing tannase enzyme isolated from *A. glaucus* to detect the appearance of certain chemical bonds. The obtained spectra for both cases are presented in Fig. 6.

Sodium alginate beads (3%) exhibited absorption bands at 3252.06 cm^− 1^ which can be due to presence of hydroxyl group (-OH), 1591.25 cm^− 1^ and 1415.29 cm^− 1^ which may refer to asymmetric and symmetric stretching vibration of COO groups, respectively. Another absorption band was shown at 1080.54 cm^− 1^ which may represent the elongation of C-O groups (Fig. 6a). The sample of Na alginate carrying the partially purified tannase from *A. glaucus* showed absorption bands at 3341.08, 1120.5, 1079.75 and 605.72 cm^− 1^. These results indicate the existing of both O-H stretch rates in the intermolecular hydrogen bonds pattern, C-C and C-O stretches in the benzene structure bonds.

### Reusability of immobilized tannase on Na-alginate

**Fig. 7:**
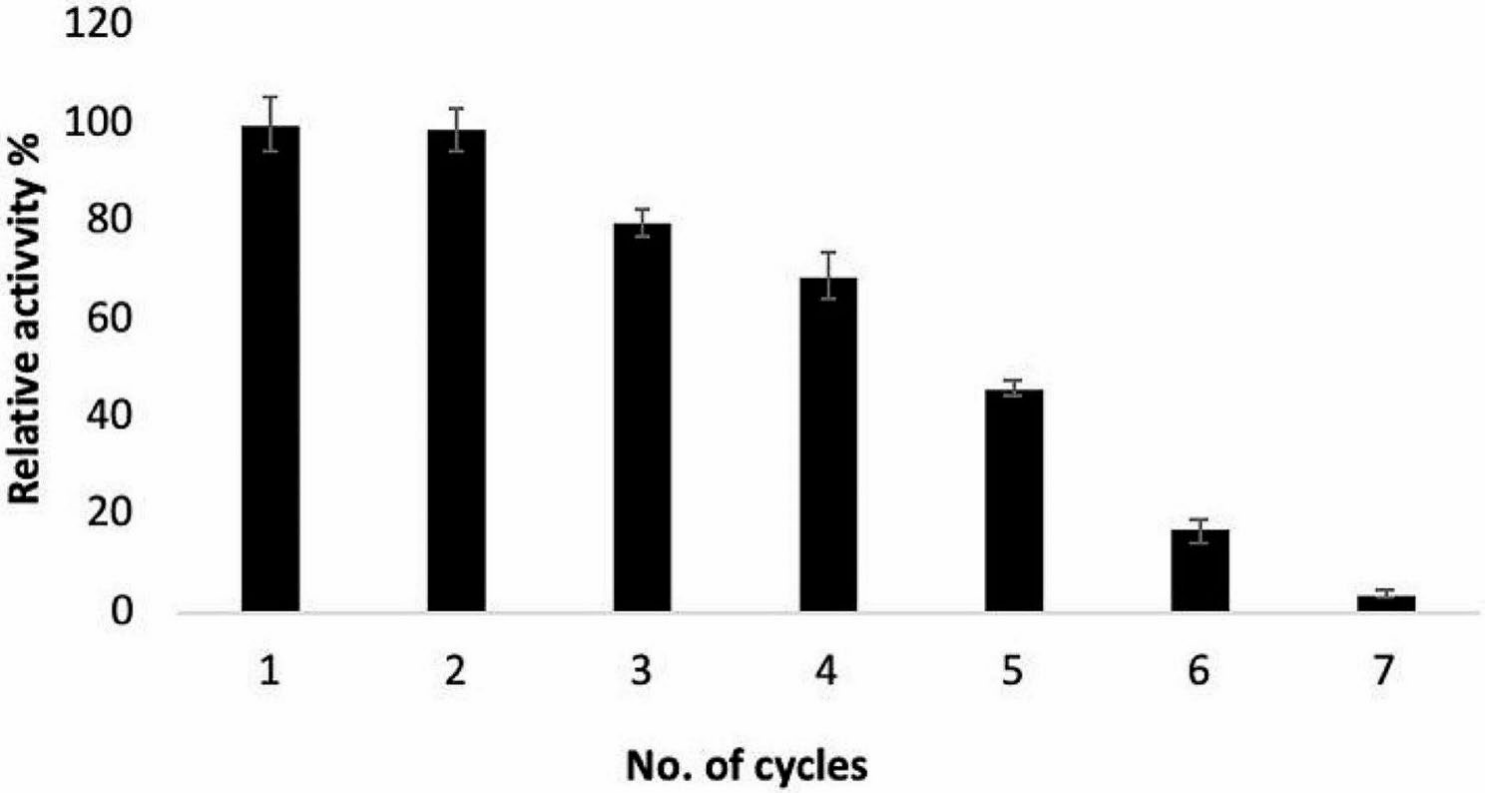
Reusability test of the immobilized *A. glaucus* tannase at repeated enzyme reactions

To evaluate the reusability of immobilized *A. glaucus* tannase, seven cycles were carried out by repeating use the Na-alginate beads after washing it well after each reaction. As shown in Fig. 7, the immobilized enzyme kept 70% of its initial activity after four reuses. The activity of immobilized enzyme was decreased by about 12% after each consecutive use till the five cycles.

### Bioconversion of tannins to gallic acid by immobilized *A. glaucus* tannase 

The efficiency of immobilized *A. glaucus* tannase for conversion of tannic acid to gallic acid was studied at two different temperatures (30 °C and 40 °C). The residual tannic acid and gallic acid produced were estimated in all samples. Results showed that the efficiency of immobilized enzyme for bioconversion of tannic acid to gallic acid was higher at 40 °C than that at 30 °C (Table 3). In addition, the highest production of gallic acid (44.80 mg/ml) was obtained at 40 °C when the tannic acid concentration was 50 g/l. Moreover, the immobilized tannase can be reused for more than three cycles in the bioconversion process with the same efficiency (data not shown).

**Table 3:** Bioconversion of tannins by immobilized *A. glaucus* tannase

Temperature	Initial tannic acid conc. (g/l)	Gallic acid conc. (mg/ml)	Residual tannic acid conc. (mg/ml)	Total tannin content (mg/ml)	Tannin degradation (%)
**30 °C**	50	35.89	26.77 ± 1.03	33.6	20.32 ± 1.21
	100	26.79	73.07 ± 2.07	89.6	18.45 ± 0.53
	200	20.77	151.14 ± 5.40	179.2	15.66 ± 1.04
**40 °C**	50	44.80	22.4 ± 0.30	33.6	33.33 ± 1.64
	100	37.20	60.48 ± 1.38	89.6	32.50 ± 0.60
	200	21.84	87.36 ± 1.08	179.2	20.66 ± 0.55

### Bioremediation of heavy metal ions using*A. glaucus*biomass

**Fig. 8:**
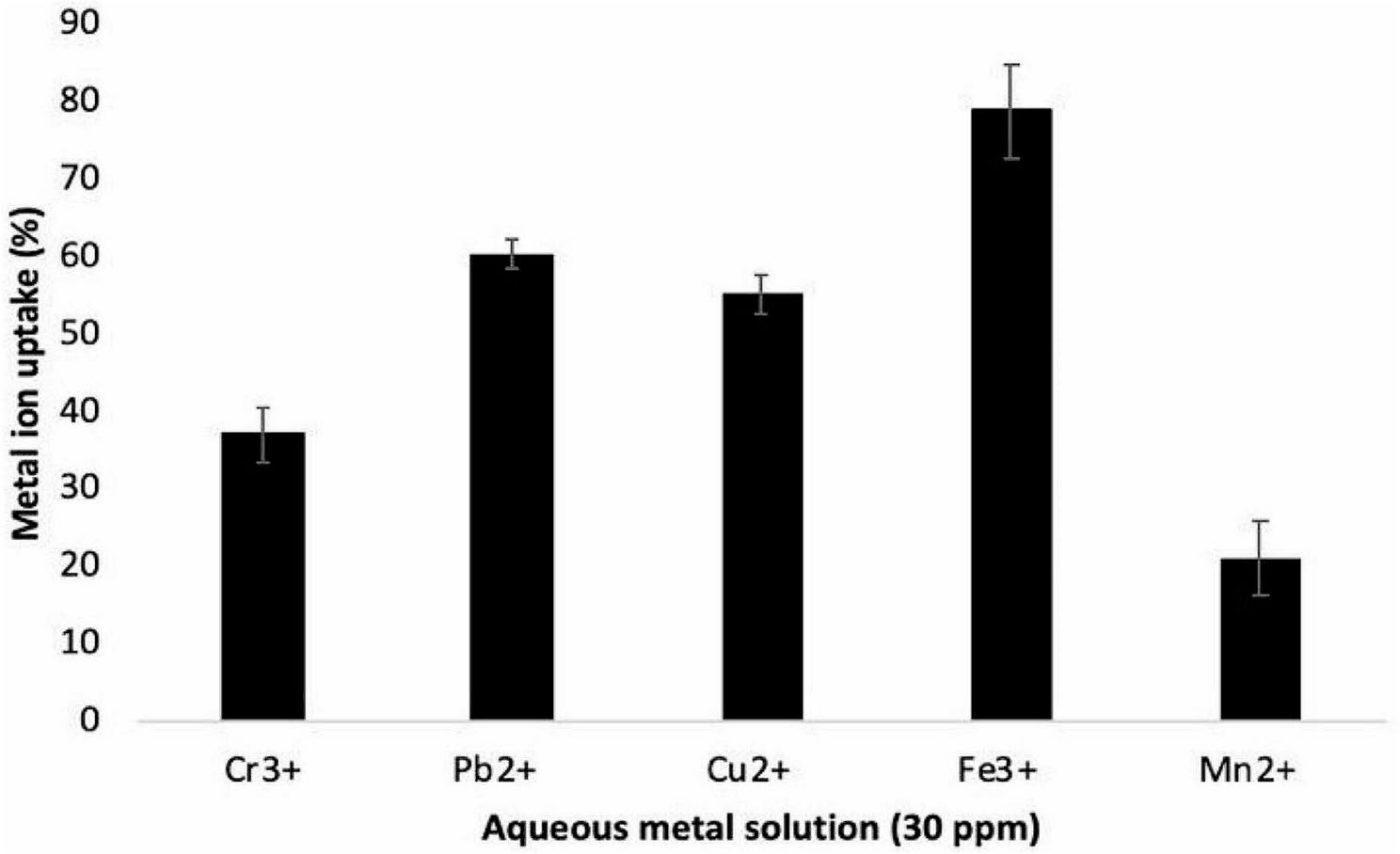
Metal ions uptake by *A. glaucus* biomass

The utilization of *A. glaucus* biomass as a biosorbent for the bioremediation of heavy metal ions, Cr^3+^, Pb^2+^, Cu^2+^, Fe^3+^ and Mn^2+^, was investigated in order to maximize the benefit of the bioprocess. Therefore, the fungal waste biomass was used to remove some heavy metals from aqueous solution. As shown in Fig. 8, the use of fungal biomass resulted in the bioremediation of heavy metal ions from aqueous solution (30 ppm) with the remediation percentage of 37.20, 60.30, 55.27, 79.03 and 21.13 for Cr^3+^, Pb^2+^, Cu^2+^, Fe^3+^, and Mn^2+^ respectively.

### Effect of live and dead fungal biomass on Cr^3+^ uptake

**Fig. 9:**
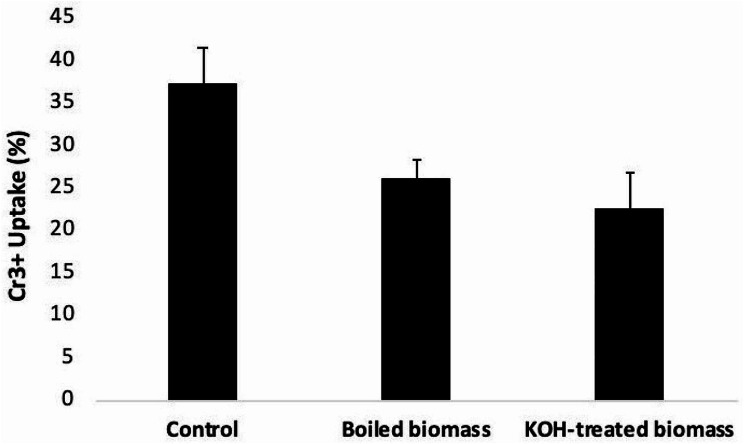
Cr^3+^uptake by *A. glaucus* biomass. Living biomass ‘control’ and dead biomass (by boiling and by KOH-treatment

Since the previous biosorption experiments were based on the living fugal biomass, thus, the impacts of using dead fungal biomass (KOH-treated and boiled biomass) compared to the living biomass, as control, on the biosorption efficiency of Cr^3+^ was investigated. Results revealed that the biosorption process of Cr^3+^ was higher when the living fungal biomass was used, indicating that fungal biomass’s uptake was dependent on cellular activity. The Cr^3+^ uptake percentage using living biomass was 37% which exceeded that of KOH-treated and boiled biomasses (26.03% and 22.60%, respectively) (Fig. 9).

### Cr^3+^ desorption and reloading of fungal biomass

The experiment was designed to recover the sorbed metal ions from fungal biomass by desorbing agent and reuse of the regenerated biomass in metal ions uptake. An efficient Cr^3+^ desorption using 0.1 N HCl as desorbing agent was noticed. Since the initial loaded Cr^3+^ on the fungal biomass was 11.15 ppm and the recovered Cr^3+^ from the fungal biomass was 5.29 ppm, thus, Cr^3+^ desorption using 0.1 N HCl was expressed as 47.44% Cr^3+^ uptake. This process was repeated for three cycles where the uptake of Cr^3+^ was decreased from 1st Cycle to 3rd cycle by (26, 13.60, 2.26%, respectively).

## Discussion

The main objective of this study was to exploit the potential of tannase from *A. glaucus* in removing tannins and fungal biomass in the bio-removal of heavy metals for treating industrial effluents like those from tanneries. Partial purification of tannase from *A. glaucus* was achieved by ammonium sulfate precipitation at saturation of 80%. The purification resulted in approximately five folds purification relative to the crude protein and substantial increase in the specific activity (from 4.1 to 19.76 U/mg). Partially purified tannase from *A. glaucus* with 66.67% yield which was higher than that reported for tannases purified from *Aspergillus niger* with 18.40% yield [15]. In addition, fractional precipitation of the *Paecilomyces variotii* culture filtrate with ammonium sulfate resulted in 78.7% yield [48]. A significant reduction in the tannic acid content of beer and orange juice was achieved by tannase purified from *Klebsiella pneumoniae* with a 9.5% yield. Indicating that tannase may be used to enhance the quality of fruit juices and beverages [2].

The biochemical studies were investigated to reveal some of the physiochemical properties of tannase that might be significant in its industrial applications. The characterization studies indicated that optimal activity of tannase from *A. glaucus* was recorded by acidic pH values (4.0–6.0) with maximum activity at pH 5.0. Similarly, it was previously reported that tannase of fungal origin is mostly active at acidic conditions (pH 4.0–6.0) while tannase of bacterial origin is primarily active at wider pH range from 4.0 to 7.0 [49, 50]. The impact of the pH on the enzyme depends on the ionization state of the amino acids on the active site. It is happened through the conformational adaptation resulted from the ionization of different amino acids [51]. Moreover, the activity of tannase purified from *A. glaucus* was favored by acidic pH, this may be because its active sites are rich in acidic amino acids. The amino acid properties of the active site have been reported to determine pH-dependent enzyme activity [52]. Accordingly, Govindarajan, R. K., et al. reported that acidic pH (pH 6.0) has a great value for tannase application in tanneries effluent degradation and pharmaceutical industry [53]. Thus, our results are found to be similar to earlier reports on tannases from different fungi such as *Aspergillus ruber* and *Verticillium* sp P9 [54, 55].

Studies on the effect of temperature on the activity and stability of *A. glaucus* tannase revealed that, the enzyme was optimally active at 40 °C. A gradual decrease in enzyme activity was observed at higher temperatures. High temperature could lead to the change of secondary and tertiary structures of tannase and the enzyme stability would decrease when the conformation of the protein was destroyed. In addition, The α-helix content of tannase decreased and the ratio of randomness increased at high temperatures [56]. This findings might refer to the fact that high temperatures increases the reaction velocity and also the rate of enzyme denaturation [57]. Similarly, optimal tannases activity obtained from *Penicillium notatum* NCIM 923 was reported at 40 °C [58].

Concerning temperature stability, it was found that tannase from *A. glaucus* was stable in the temperature range 30–60 °C retaining approximately 50% of its initial activity at 60 °C. Additionally, tannase from *A. glaucus* exhibited a maximal ionic stability at pH range 4.0 to 6.0 as more than 90% of the maximal activity remained after 12 h of incubation at pH 5.0. These results indicate that *A. glaucus* tannase is a stable enzyme under different physiological conditions which is considered as an added value for tannase-assisted bioprocesses performed at high temperatures. In this context, the optimal activity and stability of tannases of different origins were reported in a range of 30–60 °C, whereas fungal tannases showed higher activity and stability under different temperatures than those from bacteria and yeast [49, 59, 60].

A study investigating the effect of different metal ions on tannase activity indicated that all the tested metal ions did not impact enzyme activity, except for Fe^2+^ and Cu^2+^, which negatively affected tannase activity. Additionally, tannase from *A. glaucus* was partially inhibited by EDTA which indicates that the active site of the enzyme has amino acids contains sulfur, as it was previously reported by Da costa et al. [59]. In a similar fashion, Chhokar, Vinod, et al. reported that the purified tannase from *Aspergillus heteromorphus* MTCC 8818 was inhibited by several metals such as Hg^2+^ and Ag^+^ including also Fe^2+^ and Cu^2+^ [61].

In this study, the immobilization technique was selected based on the immobilization efficiency and stability of the immobilized enzyme. Immobilization of tannase was achieved using different concentrations of Na alginate compared to chitosan as immobilization carriers. The results indicated that the 3% Na alginate was the best carrier for tannase immobilization among others with an immobilization efficiency of 75%. Lower immobilization efficiency of tannase was noticed by increasing the Na alginate concentrations above 3% which could be interpreted by lack of uniform pore size due to the high viscosity of the tannase-alginate mixture, as it was similarly reported by Keerti et al. [62]. Increased Na alginate concentrations in beads have the potential to improve stability, but they may also limit the diffusion of enzymes, which would lower the reaction rate. Aharwar and Parihar (2023) reported a decrease in tannase activity of *Talaromyces verruculosus* immobilized in alginate, may be due to the active site blockage [63]. Na alginate proved to be the best support to immobilize *A. glaucus* tannase, with an immobilization efficiency of 75%, which was higher than that of immobilized *Aspergillus niger* tannase (36.8%) [64] and *Penicillium variable* tannase with the immobilization yields of 69% [65]. Yao et al., (2014) found that tannase encapsulation in calcium alginate was the best immobilization method with 62%, while Amberlite IRC 50 was the least efficient support (5%, immobilization yield) [66].

Scanned electron microscopy and FT-IR spectroscopy were used as modern and precise tools to study the structure of many samples used in biological systems [67]. Other papers similarly examined the absorption spectra of the purified tannase. For instance, Govindarajan et al., 2019 have been reported similar absorption bands for their purified tannase at 3440.91, 1466.68, 1054.60 and 699.07 cm^− 1^ by which they indicated the ability of tannase isolated from *Bacillus subtilis* KMS2-2 to degrade tannic acid as substrate into simple ester molecule [53].

The enzyme reuse is the main advantage obtained from the immobilization process. The possibility of reusing the immobilized tannase-alginate preparation was assessed for seven cycles. The results revealed that the immobilized tannase retained about 70% of its initial activity after the fourth cycles, whereas the activity of immobilized enzyme was then decreased by the number of enzyme reuse. This result can be interpreted by the enzyme inactivation occurred upon the enzyme loss from the gel due to diffusion effects as well as the multiple use and of enzyme [68]. The operational stability of the immobilized tannases is one of the most important factors in industrial applications [66]. Srivastava A, Kar R. reported that *Aspergillus niger* tannase immobilized on Na-alginate can hydrolyze 68.8% of tannins in fresh aonla juice in the first run, but can only hydrolyze 37.7% of tannins after the second run [69]. Also, tannase immobilized on Ca alginate beads had good operational stability when it hydrolyzes 56% of tea tannins from a green tea infusion after six cycles [66].

One of the important role of the tannase enzyme is obtaining gallic acid through degradation of tannins from environmentally hazardous tanneries [1]. Gallic acid formed can be directly used as a feed additive and pharmaceutical applications [70]. The efficiency of immobilized tannase of *A. glaucus* for bioconversion of tannins and production of gallic acid was higher at 40 °C than 30 °C. The high enzyme activity remarked at 40 °C can be interpreted by incidence of higher substrate solubility at higher temperatures which in turn accelerate the reaction speed. On the other hand, it was found that the efficiency of bioconversion of tannic acid to gallic acid was inversely proportional to the tannic acid concentrations. This finding can be explained by lowering of the mass transfer and substrate diffusion at high tannic acid concentration [46]. Our findings demonstrated that the immobilized tannase can be used more than three cycles for bioconversion of tannic acid to gallic acid with high efficiency compared to the free tannase from *A. glaucus* [38].

Recently, different microorganisms showed a high capability of heavy metal biosorption from wastewater [71, 72]. Therefore, we further investigated the biosorption of heavy metals such as Cr^3+^ using living and dead *A. glaucus* biomass. To obtain dead biomass of *A. glaucus*, cells were boiled in first treatment and treated with KOH as alkaline sanitizer in the second treatment. The high pH of KOH (higher than 12.0) results in cell membrane lysis and final cell lysis. Results showed a higher Cr^3+^ uptake capacity by the living fungal biomass than that obtained using dead fungal biomas. This can be explained by the fact that heavy metal biosorption including Cr^3+^ uptake by fungal biomass was dependent on cellular metabolism. Passive metal uptake, at which the metal ion sorption is non-metabolic, can happen whether cells are living or dead. In contrast, active biosorption in which the heavy metals passed through the cell membrane to the living cells requires metabolic activity of the living cells [73–75]. Live fungal biomass also has a greater adsorption capacity of heavy metals than the equivalent dry biomass due to the abundant charges on their surfaces. The biosorption process of fungal biomass might be greater, less or equivalent than that of their living counterparts [76]. In this concern, live mass of *Aspergillus* sp. had a greater biosorption of Cu ^2+^ than dead biomass [77].

This observation was in-line with former studies reported different constituents and functional groups in the fungal cell wall which are able to attract heavy metals where the ionic state of the biomass determines the effectiveness of the biosorption [78–80]. The highest uptake of Cr^3+^ by *A. glaucus* biomass is pointing out the presence binding sites on the fungal cell wall which act as active biosorbent for Cr^3+^. The applicability of the metal biosorption process is improved by (1) potential recovery of the sorbed metal and (2) potential reuse of the biomass in multiple sorption-desorption cycles. Thus, the regenerated biomass of *A. glaucus* was used for three sorption-desorption cycles of Cr^3+^. Sukumar, C., et al. reported that reuse of adsorbed metals is highly needed to diminish the metal contamination which can be evaluated by its adsorption efficiency in multiple adsorption- desorption process [81].

These findings shed the light on the potential use of tannase from *A. glaucus* in biodegradation of hydrolysable tannins producing gallic acid which could be used for pharmaceutical applications. Bioremediation of heavy metals by employing biomass of *A. glaucus* will additionally help in tannery effluent treatment.

## Conclusions

This study shows off the ability of tannase enzyme produced by a local fungal isolate, formerly identified as *A. glaucus*, in the bioremediation of tanneries containing heavy metals and tannin. Tannase enzyme was further subjected to partial purification by 80% ammonium sulfate saturation. The partial purified enzyme was characterized where found the optimum pH and temperature were 5 and 40 °C, respectively. Furthermore, the purified enzyme was active with more than 50% of its activity even at raised temperature as 60 °C which added a high-value for the enzyme usage in industrial bioprocesses require at high temperature. This can be considered as an additional advantage, since most of the processes assisted by tannase are performed at increased temperatures. Immobilized *A. glaucus* tannase was able to bio-convert tannic acid to gallic acid at 40 °C with tannic acid concentration up to 50 g/l. Using *A. glaucus* biomass at bioremediation of heavy metal from aqueous solution was effective where uptake percentages of (Cr^3+^, Pb^2+^, Cu^2+^, Fe^3+^, and Mn^2+^) were (37.20, 60.30, 55.27, 79.03 and 21.13% respectively). Additionally, the fungal biomass was able to repeatedly remove Cr^3+^ using desorbing agent (0.1 N HCl) for three cycles. The practical application of the metal biosorption process would be robustly improved when an efficient recovery of the sorbed metal and regenerated biomass is evolved for use in multiple sorption-desorption processes.

## Data Availability

The local isolated fungal strain was identified as Aspergillus glaucus and has a GeneBank accession number MH205732.
